# Universal Feature Extraction for Traffic Identification of the Target Category

**DOI:** 10.1371/journal.pone.0165993

**Published:** 2016-11-10

**Authors:** Jian Shen, Jingbo Xia, Shufu Dong, Xiaoyan Zhang, Kai Fu

**Affiliations:** 1 Institute of Information and Navigation, Air Force Engineering University, Xi’an, Shaanxi, China; 2 Tan KahKee College, Xiamen University, Xiamen, Fujian, China; National University of Defense Technology, CHINA

## Abstract

Traffic identification of the target category is currently a significant challenge for network monitoring and management. To identify the target category with pertinence, a feature extraction algorithm based on the subset with highest proportion is presented in this paper. The method is proposed to be applied to the identification of any category that is assigned as the target one, but not restricted to certain specific category. We divide the process of feature extraction into two stages. In the stage of primary feature extraction, the feature subset is extracted from the dataset which has the highest proportion of the target category. In the stage of secondary feature extraction, the features that can distinguish the target and interfering categories are added to the feature subset. Our theoretical analysis and experimental observations reveal that the proposed algorithm is able to extract fewer features with greater identification ability of the target category. Moreover, the universality of the proposed algorithm proves to be available with the experiment that every category is set to be the target one.

## Introduction

Most network managers can benefit from a thorough understanding of the traffic composition. As the most effective method of network managing, traffic identification is making a significant difference in resource scheduling, safety analysis and future tendency prediction. Network operators are interested in traffic identification both in the view of network security and management [1]. Under many circumstances, network operators’ attention is mainly focused on the specific traffic category for the purposes of network security and planning.

To recognize the specific target category among the multi-classes traffic flows, some identification approaches have been proposed. The researchers mainly focused on the P2P networks which have remarkable differences from the others. Payload identification is the method that is used most frequently in traffic identification. Sen [2] suggested that the P2P workload was a good candidate for being managed with its high volume and good stability properties. In [3], a useful insight into filtering malware in P2P networks was presented: filtering downloads based on the most commonly seen sizes of the most popular malware could block a large portion of malicious files with a very low rate of false positives. Dario Bonfiglio [4–6] devised a methodology that successfully tackled the problem of Skype voice traffic identification and extended it to identify video-calls and voice calls. However, with the appearance of encrypted data, private agreement business and VPN business, these methods cannot recognize the target traffic flows effectively.

The targeted identification of the specific traffic category is always difficult to achieve. Owing to the traffic business properties and the correlationship among each other, not every traffic category can be identified accurately, especially the ones with lower proportion. The diversity of network traffic and differences among network businesses bring about the imbalance of traffic data, which may interfere the identification result because of less training of minority categories [7–10]. To combat the imbalance problem, which is the major problem of traffic identification, three kinds of approaches have been explored, which are sampling techniques, learning algorithms and feature extraction approaches.

1. The vast majority of studies on imbalanced data sets are devoted to sampling techniques. Sampling techniques aim to solve problems with distribution of the dataset. Under sampling, over sampling and the combination of them both are three representative means [11]. Under sampling balances data of different categories by deleting the samples of majority category, while over sampling realizes the same function by coping the samples of minority category. The combination of these two sampling methods are presented as selective preprocessing of imbalanced data (SPIDER) [12], which unites local oversampling of the minority classes with filtering difficult instances from the majority classes.

2. Learning algorithms solve the imbalance problem by optimizing the performance of machine learning on unseen data. One-class learning methods combat the over-fitting problem that occurs with most classifiers by learning just with positive data points but not any other background information [13]. However, it is also believed that the results from a classifier trained only with positive data will not be as good as those with both positive and negative data [14]. Ensemble methods create a series of classifiers all applied to the same dataset. In many cases, the performance of ensemble is much better than that of any individual classifier alone [15]. Cost-sensitive learning methods try to maximize a loss function associated with a dataset that favors the minority class instead of maximizing the overall accuracy of predictions. The learning performance is critically associated with the chosen cost matrix [16].

3. Feature extraction approaches have become the center of focus on resolving the imbalance problem of identification in the last decade. The goal of feature extraction is to select a feature subset to realize the dimensionality reduction. Many researchers have been working on the same target [17–26]. Above all the dimensional reduction approaches, feature extraction is the only one that can combat the imbalance problem of identification alone in high-dimensional datasets [27]. Forman [28] examined the classification ability of feature selection metrics on a number of text sets. The features were selected by different metrics to train linear SVMs and evaluated their performance with several parameters. In [29], a method of iterative feature extraction was presented, which utilized some successful feature selection methods, such as information gain and χ2 statistics, to iteratively extract features and perform clustering.

The methods above can enhance the accuracy and equilibrium of identification in general. However, the improvement of identification performance of every category cannot be guaranteed by one algorithm alone, and the improvement is always followed by degradation of majority classes’ performance and high computational complexity. Based on these observations, it can be seen that the existing methods are just designed to identify the specific target category, regardless of the universality of identification.

This article suggests the application of feature extraction approach in imbalanced traffic database to combat the problems of non-equilibrium and high dimensions. Subset with the highest proportion (SHP) based algorithm of feature extraction is proposed to identify the target category. We put forward the divide and select strategy to compress the data space to a smaller data subset which is more representative to realize the identification of the target category. Then, a novel feature extraction model of two stages is also presented to reduce the feature dimensions of traffic database. On the base of primary feature extraction, we distinguish the target category from the interfering one, which is confirmed according to the confusion matrix, to improve the identification accuracy. One of the advantages of SHP algorithm is that it can precisely identify any target category that is appointed, the performance of identification will not be affected by the distribution of the target category. In addition, fewer features are extracted to realize the identification of the target category with better performance. Moreover, only part of the database is applied to the procedure of feature extraction, which can simplify the process of identification at the level of data amount. In general, the identification performance of the target category in imbalanced traffic can be improved to a great extent by the proposed algorithm. To explore the contribution and universality of SHP algorithm, the experiment took every category into account as the target one for identification.

The rest of the paper is organized as follows. Section 2 provides the summary of the characteristics of traffic flows and analyzes the challenges in the identification of imbalanced traffic flows. We present the idea of universal feature extraction for targeted traffic identification in Section 3. The 4th section analyses experiment results obtained from different feature extraction approaches. Finally, section 5 draws concluding remarks.

## Challenges of Traffic Identification

### Non-equilibrium of Network Traffic Distribution

Network traffic has the property of non-equilibrium in space distribution. Based on a series of researches, it is found that 10 percent of the IP addresses contribute 90 percent of traffic [30]. Moreover, the businesses of these IP users are not fixed relatively, and the traffic of different businesses varies a lot in quantity. As a result, network traffic has imbalanced distribution in space.

This paper applies Moore database to the experiment [31]. The database contains 12 traffic categories, which are WWW①, MAIL②, FTP-CONTROL③, FTP-PASV④, ATTACK⑤, P2P⑥, DATABASE⑦, FTP-DATA⑧, MULTIMEDIA⑨, SERVICES⑩, INTERACTIVES⑪ and GAMES⑫. There are 203355 flows with 248 features in the database.

**Fig 1:**
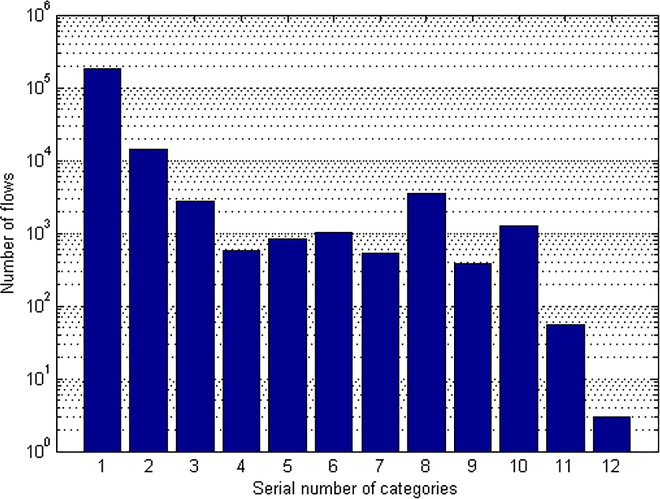
Quantity of different traffic categories.

Fig 1 shows the extreme non-equilibrium of traffic distribution. Most of the traffic concentrates in WWW① and MAIL② categories. However, there are only three flows of GAMES⑫ which takes the proportion of less than 0.002%. The result shows evident gathering phenomenon of traffic categories. To deal with the challenge, many learning algorithms have been proposed as mentioned in the section of Introduction.

### Non-equilibrium of Network Traffic Identification

The data of minority categories is likely to be misclassified in the process of identification because of the non-equilibrium of data distribution. Most algorithms are designed to classify the majority categories to achieve higher overall accuracy. As a result, the minority categories are constantly ignored.

**Fig 2:**
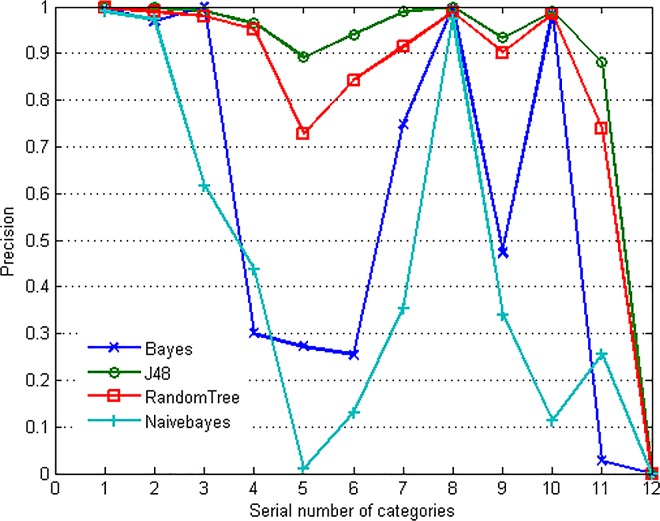
Identification precision of different categories.

Bayes, J48, Random Tree and Naivebayes classifiers are applied to traffic identification separately. The result is shown in Fig 2. It can be seen that the identification accuracy of ATTACK⑤, MULTIMEDIA⑨, INTERACTIVES⑪ and GAMES⑫ is invariably lower than the other categories. Referring to Fig 1, it can be seen that these four categories belong to the minority ones, while the categories with higher proportion are identified more accurately. The experiment shows that the non-equilibrium property of traffic can affect the identification accuracy of different categories directly. It can also be seen that some minority classes have high identification accuracy, which means the inherent property of the traffic flows is also a contributory factor. To deal with this challenge, Zhong analyzed the results of three resampling methods for handling two-class imbalance of P2P traffic traces [32]. Paper [33] presented ensemble classification algorithm embedded cost-sensitive learning to improve classification accuracy on an imbalanced dataset. Jin also built a training set using resampling method for traffic classification [34].

### Concept Drift

Network traffic has the phenomenon of concept drift in time distribution, which is supposed to be one of the most important network traffic properties. Carey Williamson [35] suggested that network traffic structure was not random and its distribution was affected by the business behavior of users in application layer. Jeffrey C. Mogul [36] pointed out the fact that most of the data in network belonged to the process that had just sent or received packages lately, and the phenomenon of concept drift even existed in microsecond range. Besides, the impulsiveness of traffic also leads to traffic gathering. Therefore, traffic flows of the same category are supposed to be relatively concentrated in a specific period of time.

**Fig 3:**
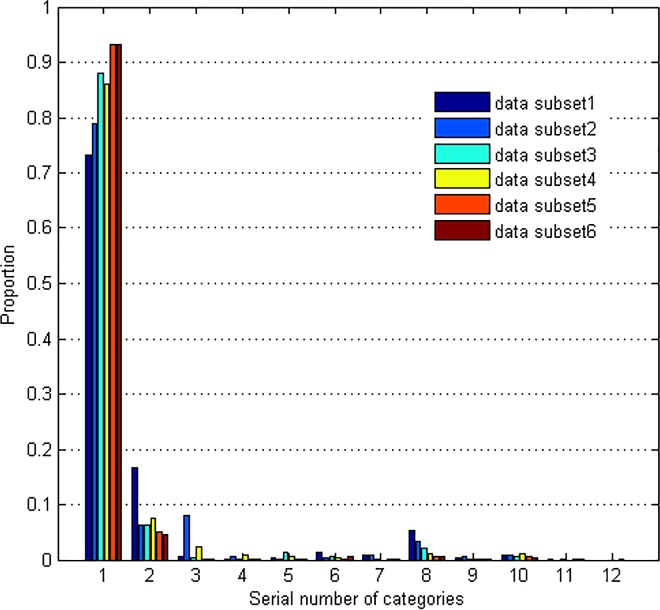
Proportion of 12 categories in different data subsets.

To compare the proportion of 12 categories in different time intervals, we divide the database into 6 data subsets according to the arriving time of flows. Fig 3 shows that the distribution of all the 12 categories in the 6 data subsets is quite different from each other, especially the minority ones. It can be seen that the traffic flows of minority categories mainly concentrate in some specific subsets, while seldom appear in the others. And the category which account for a low proportion of the subset may become majority category in another subset. To overcome this problem, Street proposed a method to retiring old classifiers one at a time [37]. Wang assigned weights to classifiers proportional to their accuracy on the most recent data block [38]. Chu viewed the choice of weights as an optimization problem and used logistic regression to settle it [39].

## Algorithm Description

We believe that no algorithm can be considered a silver bullet for identifying traffic flows of every category. From the analysis above, it can be seen that every category is always concentrated in a specific time interval, where traffic flows of the target category are more likely to be identified accurately. As one of the most effective methods of combating the class imbalance problem, feature extraction is applied to identifying the traffic flows of the target category, regardless of its proportion.

### Divide and Select

We propose the divide and select strategy to simplify the processing procedure on the level of data. Moreover, the strategy can determine the concentrated region of the target category, and the identification on the concentrated region can be more representative. The divide and select strategy parts the database *T* into several subsets {*T*_*1*_, *T*_*2*_…*T*_*n*_}, and then selects the target subset, which has the highest proportion of the target category, based on the detection of all the subsets. From the analysis above, it can be seen that the distribution of minority categories is relatively concentrated in a certain subset. Comparing to the original database, the target data subset has higher proportion of the target category, which will benefit the performance of identification. As a result of the divide and select strategy, the features extracted from the target subset are more representative for the target category.

### Primary Feature Extraction (PFE)

The traffic flows have a large number of features which may increase the computational complexity and training time. To take Moore database that is mentioned above as an example, 248 features are chosen as the attributes of traffic flows, and it will take enormous amount of time to deal with the traffic data of 248 dimensions. Feature extraction can realize dimensionality reduction and compress the quantity of features into a smaller range. On one hand, feature extraction releases the pressure of processing the multi-dimensional data. On the other hand, the method can eliminate the redundant and defective information among the features and enhance the identification performance.

In the procedure of traffic identification of the target category, the fact that accurate identification of the target category alone is not enough, but the other flows not being misclassified to the target category should also be promised. The paper uses correlation-based feature selection (CFS) estimating algorithm to extract the feature subset *F’* which can be used to classify all the categories of the database in general. CFS algorithm tries to find a subset of features not only to lower the dimensionality of the dataset but also to improve the classification accuracy. CFS defines a merit for each selected subset of features. The merit is based on the hypothesis that a promising subset of features involves those features that are uncorrelated or less correlated to each other while they are highly correlated to the class label. The merit is mathematically defined as formula (1).

$$M=\frac{kr_{cf}}{\sqrt{k+k(k-1)}r_{ff}}$$ (1)

Where *M* is the heuristic merit of a feature subset containing *k* features, *r*_*cf*_ is the mean feature-class correlation and *r*_*ff*_ is the average feature*−*feature correlation [40].

However, there are still some features, with redundant information or no contribution to the identification of the target category. So the feature subset *F’* should be refined to a smaller and more representative collection. We extract features according to F1-measure of the target category which can equally weigh precision and recall.

$$F1[f(n)]=\frac{2*Precision[f(n)]*Recall[f(n)]}{\text{Precision}[f(n)]+Recall[f(n)]}$$ (2)

The first extracted feature should be the one with the maximum of F1-measure of the target category.

$$f_{1}=\arg\;\underset{f(n)}{\max}\{\text{F}1[f(n)]\},n\in [1,N]$$ (3)

where N is the number of features extracted by CFS. From formula (3), it can be seen that the first extracted feature is the one with the greatest identification ability. In the procedure of the following feature extraction, the next extracted feature should also be the one that performs the best on F1-measure, together with the features that have already been extracted. All the following extracted features should meet the requirement below.

$$f_{x}=\arg\;\underset{f(n)}{\max}\{\text{F}1[\overset{x-1}{\underset{i=1}{\cup }}f_{i}\cup f(n)]\},n\in [1,N]$$ (4)

where x is the extracted feature ordinal. With the increasing of extracted features, the performance of the classifier will be improved evidently and the value of F1-measure will be promoted as well. Primary feature extraction keeps selecting features from the target subset until the value of F1-measure stops increasing. From the analysis above, it is clear that the generated subset of features extracted from the feature collection is more likely to identify the target category accurately and prevent the target category from being misclassified by the others.

The method of primary feature extraction is detailed in Algorithm 1.

**Algorithm 1. Primary feature extraction**

Input: *T* is the database of network traffic, *F* is the feature collection, *C* is the collection of categories, *C*_*α*_ is the target category.

Output: *F*_*p*_ is the feature subset selected by primary feature extraction.

(1)Divide database *T* into *n* subsets, {*T*_*1*_, *T*_*2*_…*T*_*n*_}

(2)Find out the target subset *T*_*t*_ which has the highest proportion of category *C*_*α*_

(3) *F*' = *CFS*(*T*_*t*_,*F*)

(4)for *f*(*j*) ∈ *F*' do

(5) $F1[f(j)]=\frac{2*Precision[f(j)]*Recall[f(j)]}{Precion[f(j)]+Recall[f(j)]}$

(6)end for

(7) $f_{1}=\arg\;\underset{f(j)}{\max}\{\text{F}1[f(j)]\}$

(8) *F*_*p*_ = *f*_1_

(9)for *i* = 2 to m //m is the number of features of *F’*

(10) $f_{i}=\arg\;\underset{f(n)}{\max}\{\text{F}1[\overset{i-1}{\underset{j=1}{\cup }}f_{j}\cup f(n)]\}$

(11) if F1[*F*_*p*_]≤F1[*F*_*p*_ ∪ *f*_*i*_] then

(12) *F*_*p*_ = *F*_*p*_ ∪ *f*_*i*_

(13) or else break

(14) end if

(15)end for

(16)return *F*_*p*_

### Secondary Feature Extraction (SFE)

Because of the similarity among different categories presented on features and the correlationship among the extracted features, the target category is likely to be misclassified to the other one or mistaken by another one. So the goal of secondary feature extraction is to improve the identification performance of the target category by distinguishing it from the class that interferes with the identification performance most seriously.

Firstly, the interfering category should be assigned. In multiclass prediction, the result on a test is often displayed as a two-dimensional confusion matrix with a row and column for each class. Each element of matrix A shows the number of test examples for which the actual class is the row and the predicted class is the column.

$$A=\left(\begin{array}{ccc} a_{11} & \ldots & a_{1n}\\ \vdots & \ddots & \vdots \\ a_{n1} & \cdots & a_{nn} \end{array}\right)$$ (5)

Where *n* is the number of the categories of the database, and *a*_*αβ*_ represents for the number of flows of category *α* which are misclassified as categor*y β*. We can obtain the value of *a*_*αβ*_ from the statistics of the identification result. With the help of the simulation software named WEKA [41], the value of all the elements in matrix A can be achieved more easily. We define the parameter of interaction as *I* to evaluate the probability of misclassification between two categories.

$$I_{\alpha \beta }=a_{\alpha \beta }+a_{\beta \alpha }$$ (6)

The class that interferes with the identification of category *α* most seriously is the one fulfilling Eq (7).

$$\begin{array}{l} \beta =\arg\;\underset{i}{\max}[I_{\alpha i}],i\in [1,n]\&i\neq \alpha \\ \;=\arg\;\underset{i}{\max}[a_{\alpha i}+a_{i\alpha }] \end{array}$$ (7)

Then, the paper extracts features to improve the identification performance according to the interfering category. The flows of the two categories can be extracted from the database by the sampling approach. We use CFS algorithm to extract the feature subset *F”* from feature collection *F* to distinguish the target category from the interfering one. The feature subset *F”* should also be simplified to reduce the computational complexity and avoid the interference of the redundant information among the features. In the following procedure of feature extraction, the extracted features are the ones that perform best on F1-measure of the target category, together with the extracted feature subset *F*_*p*_. The extracted features should meet the requirement below.

fx'=argmaxf(n){F1[∪i=1x−1fi'∪Fp∪f(n)]},n∈[1,N] (8)

Secondary feature extraction algorithm keeps selecting features from the feature subset *F”* until the value of F1-measure stops increasing.

The method of secondary feature extraction is detailed in Algorithm 2.

**Algorithm 2. Secondary feature extraction**

Input: *F* is the feature collection, *C*_*α*_ is the target category, *T*_*t*_ is the subset which has the highest proportion of category *C*_*α*_.

Output: *C*_*β*_ is the interfering class of category *C*_*α*_, *F*_*s*_ is the feature subset selected by secondary feature extraction.

(1)Find out the interfering category *C*_*β*_ from database *T*_*t*_

$\beta =\arg\;\underset{i}{\max}[a_{\alpha i}+a_{i\alpha }],i\in [1,n]\&i\neq \alpha$

(2) Extract the flows of categories *C*_*α*_ and *C*_*β*_ from database *T*_*t*_ as database *T*_*t*_*’*.

(3) *F*" = *CFS*(*T*_*t*_',*F*)

(4)for *f*(*j*) ∈ *F*" do

(5) $F1[f(j)]=\frac{2*Precision[f(j)]*Recall[f(j)]}{Precion[f(j)]+Recall[f(j)]}$

(6)end for

(7) $f_{1}=\arg\;\underset{f(j)}{\max}\{\text{F}1[f(j)]\}$

(8) *F*_*s*_ = *f*_1_

(9)for *i* = 2 to m //m is the number of features of *F”*

(10) $f_{i}=\arg\;\underset{f(n)}{\max}\;\{\text{F}1[\overset{i-1}{\underset{j=1}{\cup }}f_{j}\cup F_{p}\cup f(n)]\}$

(11) if $F1[\overset{i-1}{\underset{j=1}{\cup }}f_{j}\cup F_{p}]\leq F1[\overset{i}{\underset{j=1}{\cup }}f_{j}\cup F_{p}]$ then

(12) *F*_*s*_ = *F*_*s*_ ∪ *f*_*i*_

(13) or else break

(14) end if

(15)end for

(16)return *F*_*s*_

There is a special case in the process of primary feature extraction that the value of F1-measure of the target category is zero, which means the feature subset extracted by CFS algorithm doesn’t take effect in the identification procedure. Under this circumstance, we select the flows of the target category and the interfering category which is misclassified worst as the database. Then the primary feature subset is determined by CFS algorithm from the new generated database.

According to the primary and secondary feature extraction, the final feature subset *F*_*final*_ is obtained with the combination of *F*_*p*_ and *F*_*s*_.

$$F_{final}=F_{p}\cup F_{s}$$ (9)

## Experiment and Result

### Experimental Setup

Subset-based extraction techniques can either be filters or wrappers [42]. Filters use statistical tests to decide on subset scoring and can select the subset very fast. Wrappers use the tested features to build a classification model, the performance of which is applied to the evaluation of the features. Usually wrapper approaches can extract features with higher prediction performance than filters according to specific learning algorithms. However, wrapper approaches are less common than filter ones because they consume more computational resources and are often intractable for large scale problems. In the experiment, we compare the performance of SHP algorithm with wrapper, CFS-based filter and Information Gain (IG) methods respectively. Wrapper approach uses a classifier to evaluate attribute sets, and it employs across-validation to estimate the accuracy of the learning scheme for each set [41]. This paper uses the wrapper with the classifier of NaiveBayes as the competing method. And the accuracy of the classifier is used to evaluate the performance of the attribute combinations. IG evaluates attributes by measuring their information gain with respect to the class. We select the top 10 features to be the extracted feature subset as a comparison.

Moore database mentioned in section 3 is applied to the experiment. The features extracted by IG, CFS and Wrapper approaches from database *T* are {4,107,108,201,95,198,200,191,194,98}, {4,78,107,111,119,145,149,175,200} and {4,8,65,68,79,95,113,181,201,202}. Moore database *T* is divided into 6 data subsets {*T*_*1*_, *T*_*2*_…*T*_*6*_} according to the arriving time of flows. The features extracted by CFS algorithm from the 6 data subsets are shown in Table 1. The features extracted by SHP algorithm in different stages are shown in Table 2. The numbers in the tables are feature references.

**Table 1:** Features extracted by CFS algorithm.

Data subset	*F’*
***T**_**1**_*	4,50,72,91,108,155,202
***T**_**2**_*	4,72,78,108
***T**_**3**_*	4,78,109,137,148
***T**_**4**_*	4,51,109,137,180
***T**_**5**_*	4,29,78,83,137
***T**_**6**_*	4,66,78,81,137,148

**Table 2:** Features extracted by SHP algorithm in different stages.

Target category	Target data subset	PFE	SFE	SHP
**WWW**	*T*_*6*_	4,137	107	4,137,107
**MAIL**	*T*_*1*_	108,72,91,155,202,4,50	107	108,72,91,155,202,4,50,107
**FTP-CONTROL**	*T*_*2*_	72,108	107,196	72,108,107,196
**FTP-PASV**	*T*_*4*_	4,109	Φ	4,109
**ATTACK**	*T*_*3*_	4,107,108,174	179,200,193,99	4,107,108,174,179,200,193,99
**P2P**	*T*_*1*_	4,155,202,108	19,74	4,155,202,108, 19,74
**DATABASE**	*T*_*1*_	4	91	4,91
**FTP-DATA**	*T*_*1*_	108,91,155,72	8,124	108,91,155,72,8,124
**MULTIMEDIA**	*T*_*2*_	4	8	4,8
**SERVICES**	*T*_*4*_	98,107	Φ	98,107
**INTERACTIVE**	*T*_*6*_	4,14,71,107,167	108,199,19	4,14,71,107,167,108,199,19
**GAMES**	*T*_*5*_	4,29	99	4,29,99

The experiment adopts Naivebayes as the classifier. The 10-fold cross-validation method is used to estimate the data. The result takes the average of the experiment which is conducted ten times.

### The Performance of SHP Algorithm

The experiment identifies the target category from database *T* with all the features as the baseline and feature subsets extracted by the methods of IG, CFS, Wrapper, and SHP. Every one of the 12 categories is set as the target one to be classified from the others. To compare the performance of different methods on majority and minority classes, we choose six categories that have the highest or lowest proportion of traffic flows as representatives of all the categories. The majority classes are WWW, MAIL and FTP-DATA, while the minority classes are MULTIMEDIA, INTERACTIVE and GAMES.

**Fig 4:**
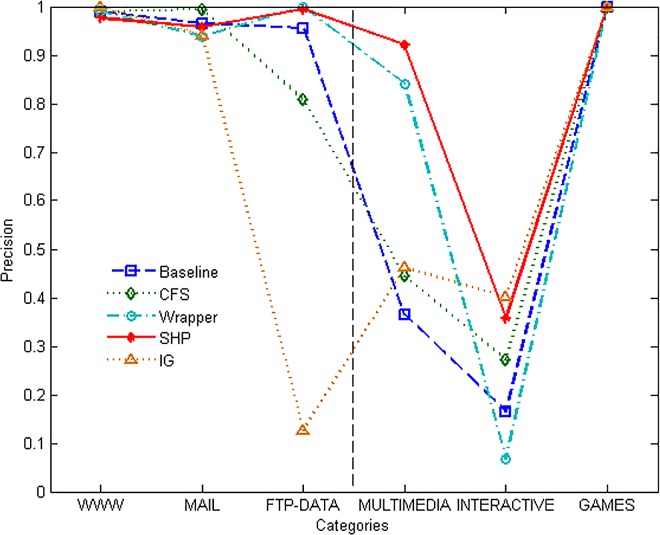
Precision of different methods.

From Fig 4 it can be seen that the identification results of the majority classes of the approaches have similar high precision except for IG. That means most of the approaches can recognize the majority target categories with great accuracy. However, they do not have similar performance on the minority classes, especially on INTERACTIVE category. Even so, the precision of identification of the SHP algorithm is higher than the others.

**Fig 5:**
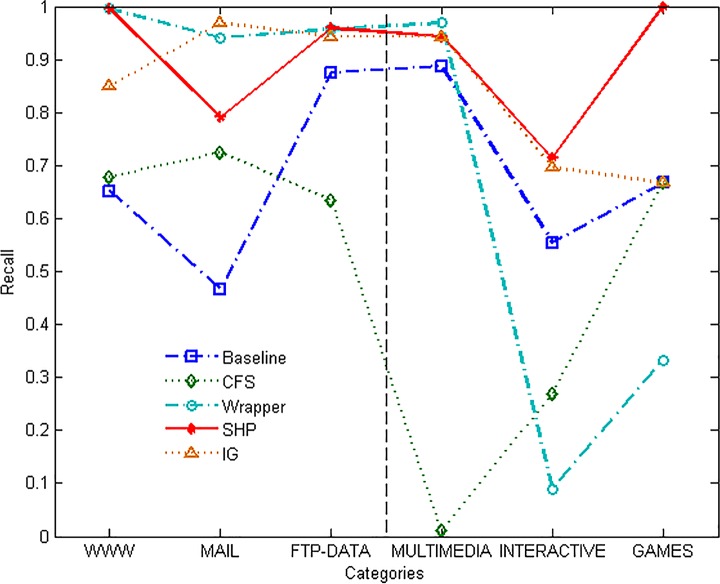
Recall of different methods.

From Fig 5, we can see that the features extracted by CFS algorithm perform worse than all the features on the parameter of recall. That is because the process of CFS algorithm does not only reduce the data dimensions, but also decreases the available information amount. Wrapper approach gets high score on the majority categories, and presents the weakness on the minority categories at the same time. SHP algorithm has similar performance as Wrapper and IG approaches on the majority categories, meanwhile outperforms all the other methods on the minority ones.

**Fig 6:**
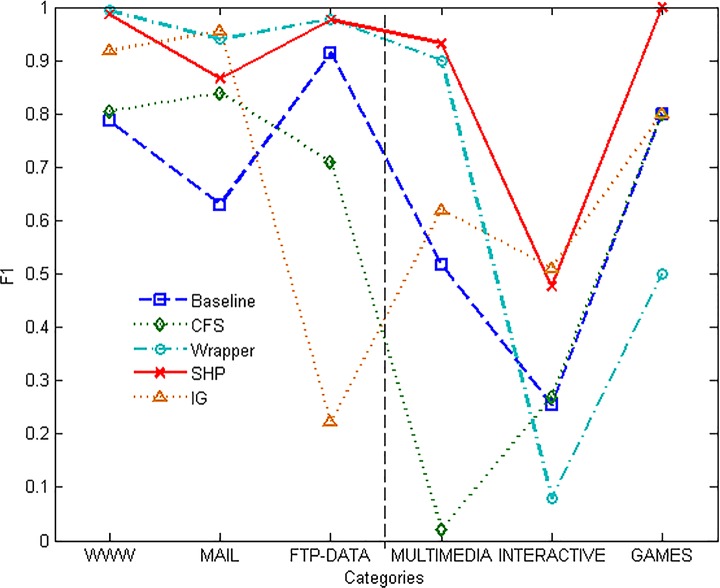
F1-measure of different methods.

Fig 6 shows the performance of F1-measure which is the comprehensive parameter of precision and recall. All the approaches present relative better performance on the majority categories than the minority ones. IG method scores more than SHP on MAIL and INTERACTIVE, but performs badly on the other categories. It can be seen that the performance of IG is not only affected by the proportion, but also the intrinsic properties of the target category. That is why the performance of IG is not stable. Except for that, SHP algorithm outperforms every other method on the parameter of F1-measure of all the chosen categories.

**Fig 7:**
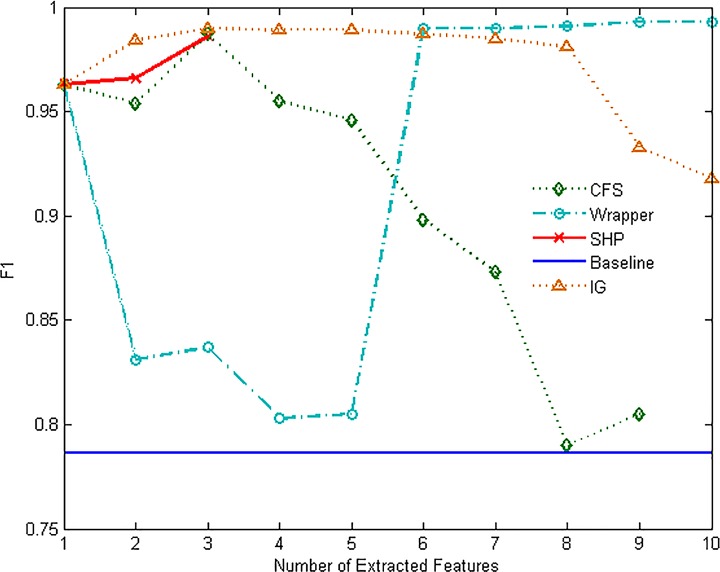
Performance of F1-measure on WWW category.

**Fig 8:**
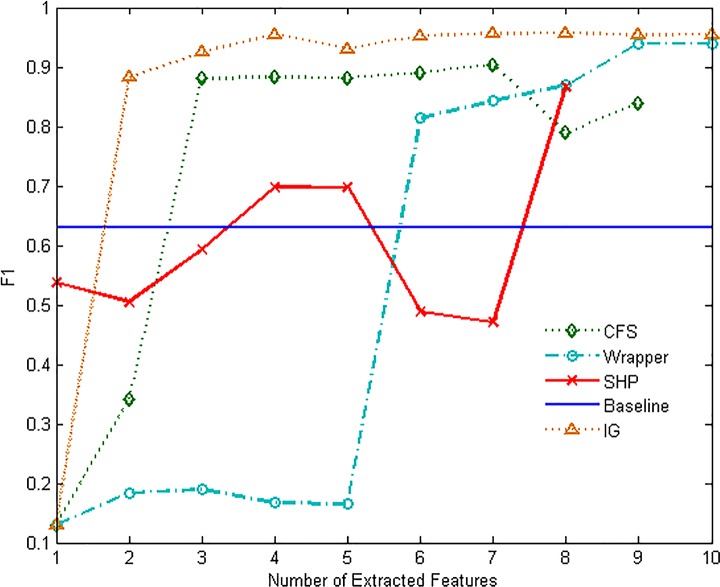
Performance of F1-measure on MAIL category.

**Fig 9:**
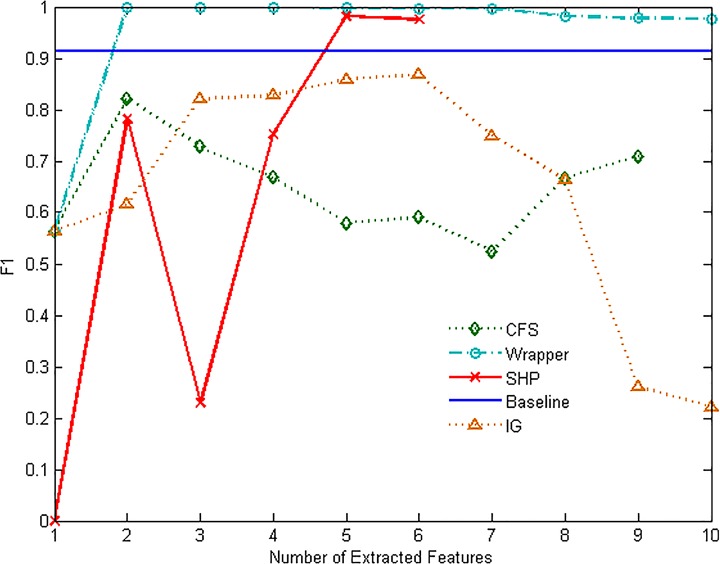
Performance of F1-measure on FTP-DATA category.

**Fig 10:**
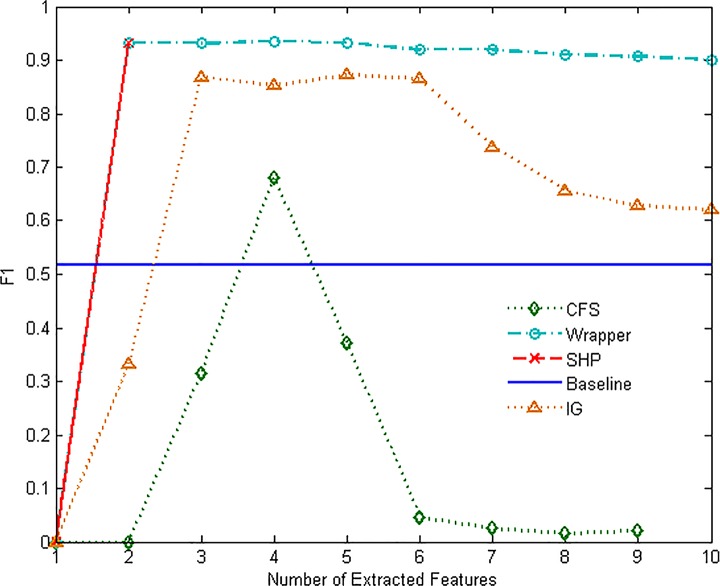
Performance of F1-measure on MULTIMEDIA category.

**Fig 11:**
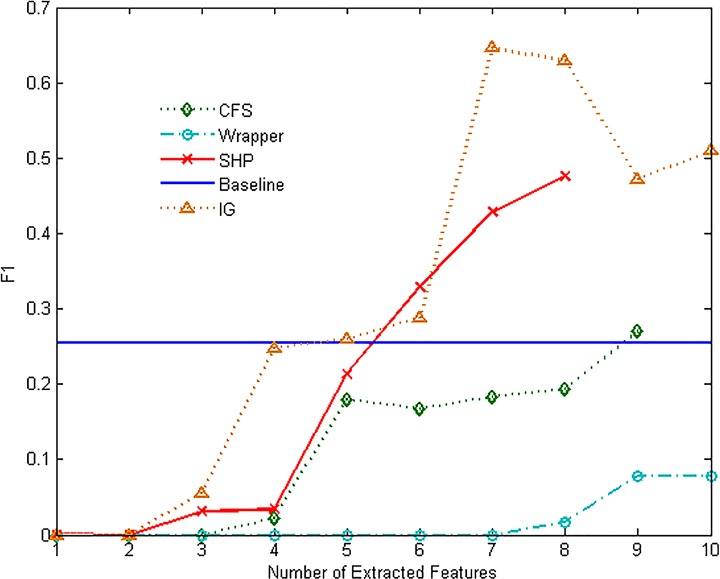
Performance of F1-measure on INTERACTIVE category.

**Fig 12:**
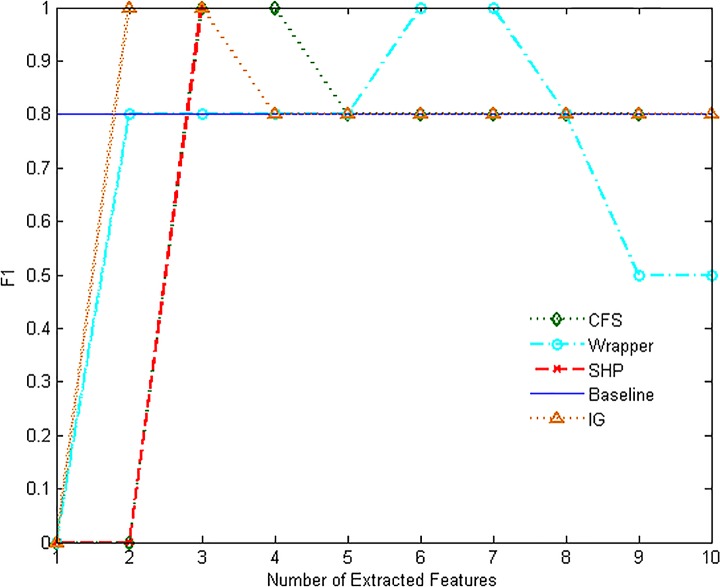
Performance of F1-measure on GAMES category.

Figs 7–12 show the performance of F1-measure of different approaches on majority and minority categories. Both of Wrapper and SHP methods show an upward trend on F1-measure with the number increasing of extracted features. CFS and IG present the differential performance according to tested categories. In some cases, IG method can get the highest score. However, in the others, the performance of IG and CFS is even worse than the baseline, which means the two methods cannot make a difference in improving identification ability in such situations. The value of F1-measure of Wrapper approach rises quickly with the increasing of features. However, when the value of F1-measure gets to the highest point, the number of extracted features still keeps increasing. As a result, the value of F1-measure stops rising or even starts to decrease. SHP algorithm can also achieve high performance of F1-measure quickly just as Wrapper approach. Moreover, the number of features stops increasing when the performance reaches the highest point. So SHP algorithm can always get the highest score with much fewer extracted features. The experiment result reveals the identification accuracy and efficiency of SHP algorithm.

Receiver operating characteristic (ROC) curve is the method of patterning to show the true positive rate (TPR) and false positive rate (FPR) of identification model. ROC curve can reflect the performance of a classifier regardless of the class distribution or error costs. The closer the curve approaches to the top left corner, the better the performance of the classifier is. To quantitatively assess the identification model, the area under the curve of ROC is adopted as the evaluating parameter. For highly imbalanced data as traffic flows, it is also a good choice to use the area under the P-R curve (PRC) as the evaluation statistic along with the area under the ROC curve.

**Fig 13:**
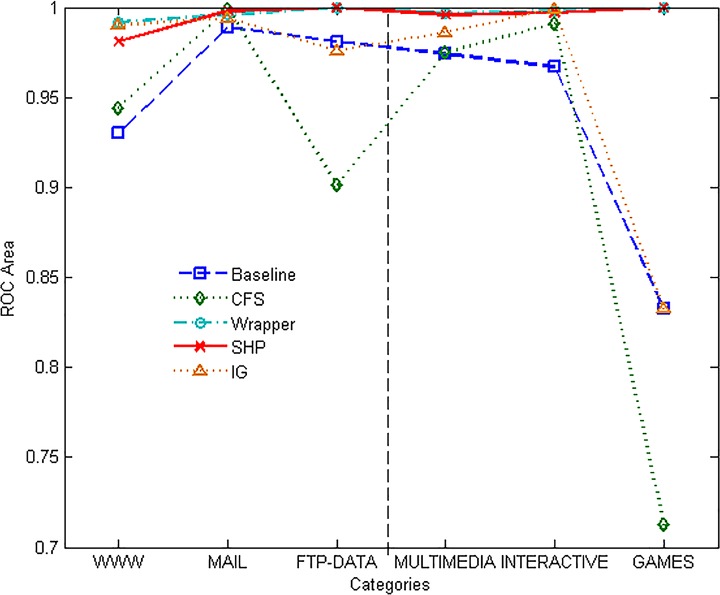
ROC performance of different methods.

**Fig 14:**
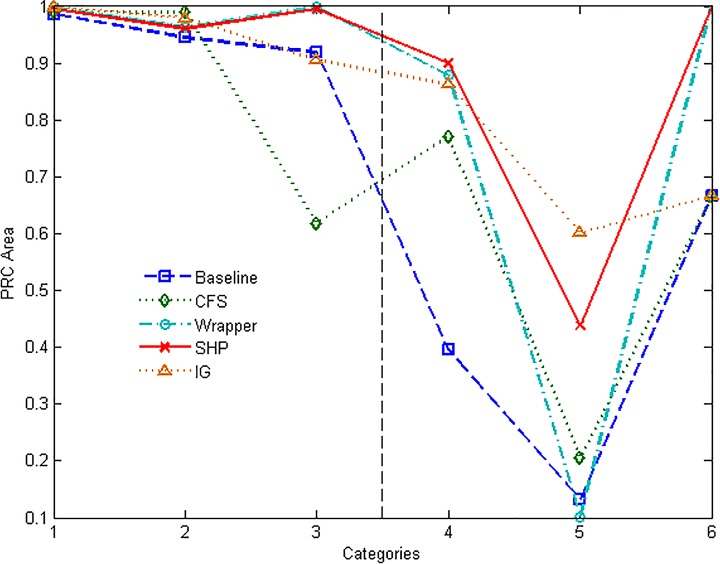
PRC performance of different methods.

Figs 13 and 14 show the ROC and PRC graphs for the chosen 6 categories. It can be seen that SHP and Wrapper approaches perform similarly and better than the others on ROC Area. The value of PRC Area of the minority category is obviously affected by its proportion. IG has good performance on INTERACTIVE category, and performs regularly on the others. Except for that, SHP algorithm outperforms the other approaches on every category.

**Fig 15:**
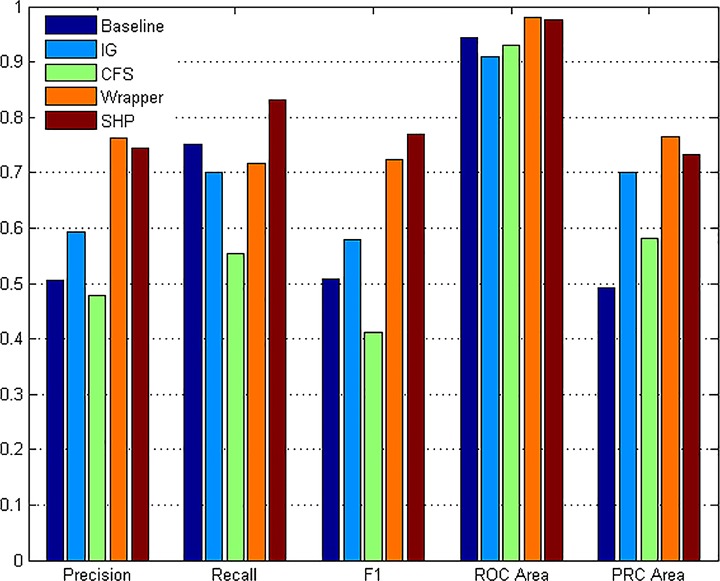
Average identification performances of different algorithms.

We conduct the experiment with each method on all the categories. The performance of each method is the average of the 12 independent tests of all the categories. The collected result is shown as Fig 15. It is obvious that the features extracted by SHP algorithm perform much better than those of IG, CFS methods and the baseline on every parameter. Compared with Wrapper, SHP algorithm shows a bit lower precision but much higher recall rate and F1-measure which presents the comprehensive identifying ability. On the parameters of ROC and PRC, the performances are nearly the same.

Table 3 shows the average number of extracted features of each method. All the three feature extraction approaches play a part in reducing the data dimensions remarkably. And SHP algorithm is the one that compressing the number of features to the least. Based on the statistical analyses, it can be seen that SHP algorithm presents the superiority in target category identification with less features.

**Table 3:** Average number of extracted features.

Baseline	IG	CFS	Wrapper	SHP
248	10	9	10	**4.5**

We choose two more databases to analyze the challenges mentioned in the second section, which are non-equilibrium of network traffic distribution, non-equilibrium of network traffic identification and concept drift. Meanwhile, we compare the performances of different feature extraction approaches on the two chosen databases to certify the applicability of SHP algorithm. The two databases *T’* and *T”* are chosen from different time sections containing traffic features of 55494 and 23801 flows respectively.

**Fig 16:**
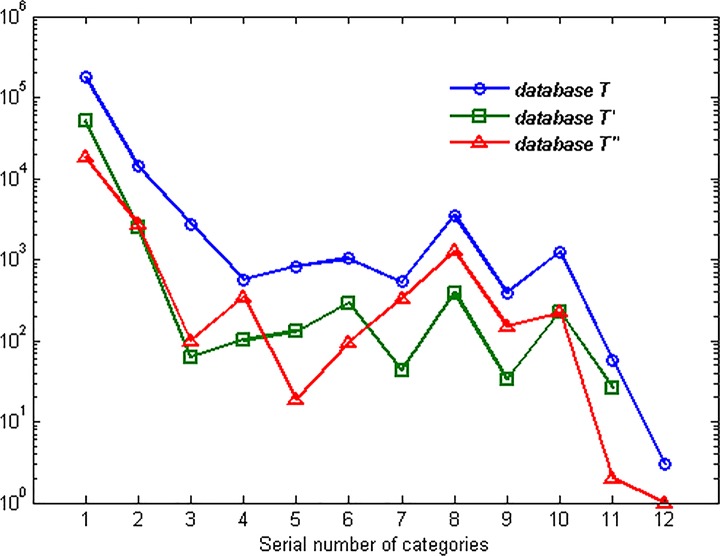
Quantity of different categories in the three databases.

**Fig 17:**
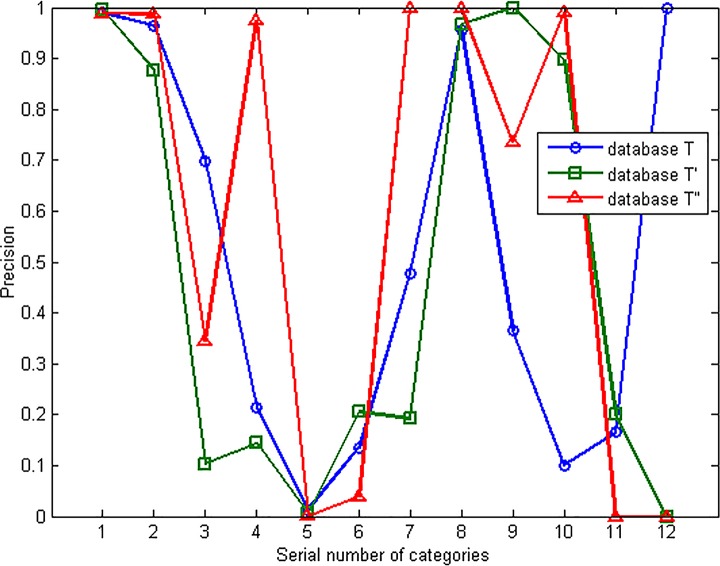
Precision of different categories in the three databases.

The components of the three databases are shown in Fig 16. The phenomenon of non-equilibrium of network traffic distribution is obvious. Most of the traffic flows are concentrated in WWW① and MAIL② categories, while some categories only take a small part of the database like INTERACTIVES⑪ and GAMES⑫. Fig 17 shows the identifying precision of the three databases with the classifier of NaiveBayes. Because of the non-equilibrium of traffic distribution, the identification precision of each category differs a lot according to the distribution.

Meanwhile, with the comparison of the same category distributed in different databases, which contain traffic flows of different time intervals, we can observe the phenomenon of concept drift as the result of the distribution of the same category keeps changing as time goes on. Above all, we can clearly see that the three challenges mentioned previously are widely existed in traffic flows.

We also conduct the experiment with each identifying method on all the categories and take the average performance of the 12 independent tests as the result shown in Table 4. It can be seen that SHP algorithm has good performance on the identification parameters of precision rata and recall rata. And it can always get the highest score on the comprehensive identification parameter of F1-measure. On the parameter of ROC area and PRC area to quantitatively assess the identification model, wrapper and SHP approaches always have the best performance. Moreover, the number of features extracted by SHP algorithm is the least among the mentioned approaches. Above all, SHP algorithm has better performance than the other methods and performs similarly on all the three databases. Besides the comparison above, it is clear that only one sixth of the data has been processed in the experiment by SHP algorithm which can speed up the feature extraction procedure significantly.

**Table 4:** Average identification performances of database *T’* and *T”*. (The best results for each algorithm are shown in bold).

	*T’*					*T”*				
	Baseline	IG	CFS	Wrapper	SHP	Baseline	IG	CFS	Wrapper	SHP
**Precision**	0.5081	0.5236	0.4792	0.5975	**0.7877**	0.6418	**0.8172**	0.5286	0.7902	0.8069
**Recall**	**0.7983**	0.6419	0.4080	0.5885	0.7505	0.7526	0.6977	0.4514	0.7485	**0.8185**
**F1**	0.5114	0.5121	0.4205	0.5929	**0.7402**	0.6315	0.7080	0.4005	0.7656	**0.7966**
**ROC Area**	0.9518	0.9169	0.9039	**0.9805**	0.9693	0.9102	0.9511	0.8192	**0.9628**	0.9354
**PRC Area**	0.5075	0.6504	0.4512	0.6308	**0.7269**	0.6375	0.8085	0.5084	**0.8710**	0.8052
**Number of features**	248	10	6	5	**4.7**	248	10	7	14	**4**

From the experiment, we can see that wrapper approach is also promising with good performance on many parameters. However, it should be mentioned that the computational complexity of Wrappers method is so high that the consuming time is intolerable during the process of experiment. From the analysis of theory, we can figure out the reason of the problem is that the computational complexity of wrapper method is *O*(2^n^), which is intractable for high-dimensional data just as traffic flows. While the computational complexity of IG, CFS and SHP is respectively *O*(n), *O*(n^2^) and *O*(n^2^).

## Conclusion

To achieve accurate identification of the target category, we have presented a feature extraction method based on the subset with highest proportion. The experiment showed that the proposed algorithm was able to extract features with great identification ability of the target category. SHP algorithm also presented the universality on feature extraction for any category, no matter it belonged to the majority or minority classes. Moreover, compared with the other feature extraction approaches, only half of the features were required for SHP algorithm on average. This property made considerable reduction on the training complexity and the consuming time of testing. As future work, we intend to investigate the combination of filter and wrapper approaches for identification of multi-classes. Future research should also realize the traffic identification based on the proposed model of two stages, and take advantages of both wrapper and filter approaches.

## Supporting Information

S1 File Database *T”*. This document contains the database *T”*.
(RAR)

## References

[1] Arthur C, Judith K, Djamel S, Carlos AK, Stenio F. Better network traffic identification through the independent combination of techniques. Journal of Network and Computer Applications. 2010 7;33(4):433–14.

[2] Sen S, Wang J. Analyzing peer-to-peer traffic across large networks. IEEE/ACM Transactions on Networking (TON). 2004;12(2):219–14.

[3] Kalfut A, Acharya A, Gupta M. A study of malware in peer-to-peer networks. Proceedings of the 6th ACM SIGCOMM conference on Internet measurement; 2006 Oct 327–6; New York, USA.

[4] Bonfiglio D, Mellia B, Meo M, Rossi D. Detailed analysis of Skype traffic. IEEE Transactions on Multimedia. 2009 1;2(1): 117–11.

[5] Zhang HL, Gu Zm, Tian ZQ. Skype Traffic Identification Based SVM Using Optimized Feature Set. 2010 4;12(2):149–8.

[6] Bonfiglio D, Mellia M, Meo M, Rossi D, Tofanelli P. Revealing Skype traffic: When randomness plays with you. ACM SIGCOMM Computer Communication Review; 2006 Aug 130–11; Kyoto, Japan.

[7] Garcia EA. He H. Learning from imbalanced data. IEEE Trans. Knowledge and Data Engineering. 2009;21(9):1263–22.

[8] Padmaja TM. Majority filter-based minority prediction (MFMP): An approach for unbalanced datasets. IEEE Region 10 Annual International Conference; 2008 Nov 1–6; Hyderabad, India.

[9] He HB, Chen S, Man H, Desai S, Quoraishee S. Imbalanced learning for pattern recognition: An empirical study. Unmanned/Unattended Sensors and Sensor Networks VII; 2010 Sep 1–7; Toulouse, France.

[10] Callado A, Kamienski C, Szabo G, Gero B, Kelner J, Fernandes S, et al A Survey on Internet Traffic Identification. IEEE Commun. Surveys & Tutorials. 2009;11(3):35–6.

[11] Alibeigi M, Hashemi S, Hamzeh A.DBFS: An effective Density Based Feature Selection scheme for small sample size and high dimensional imbalanced data sets. DATA & KNOWLEDGE ENGINEERING. 2012 11;81–82: 67–37.

[12] Stefanowski J, Wilk S. Selective pre-processing of imbalanced data for improving classification performance. Data Warehousing and Knowledge Discovery. 2008;16(5):283–10.

[13] Elkan C, Noto K. Learning classifiers from only positive and unlabeled data. Proceedings of the 14th ACM SIGKDD International Conference on Knowledge Discovery and Data Mining; 2008 Aug 213–220.

[14] Manevitz LM, Yousef M. One-Class SVMs for Document Classification. JOURNAL OF MACHINE LEARNING RESEARCH. 2001;2(2):139–16.

[15] Huang KZ, Yang HQ, King I, Lyu MR. Learning classifiers from imbalanced data based on biased minimax probability machine. Proceedings of the 2004 IEEE Computer Society Conference on Computer Vision and Pattern Recognition; 2004;2:558–6.

[16] Masnadi-Shirazi H, Vasconcelos N. Asymmetric boosting. Machine Learning, Proceedings of the Twenty-Fourth International Conference. 2007:609–11.

[17] Hou C, Nie F, Tao D. Discriminative Vanishing Component Analysis. AAAI. 2016: 1666–1672.

[18] Hou C, Nie F, Li X, Li X, Wu Y. Joint embedding learning and sparse regression: a framework for unsupervised feature selection. IEEE Transactions on Cybernetics, 2014, 44(6):793–804. 10.1109/TCYB.2013.2272642 23893760

[19] Nie F, Xiang S, Jia Y, Zhang C. Trace ratio criterion for feature selection// National Conference on Artificial Intelligence. AAAI Press, 2008:671–676.

[20] Gui J, Sun Z, Cheng J, Ji S. How to Estimate the Regularization Parameter for Spectral Regression Discriminant Analysis and its Kernel Version? IEEE Transactions on Circuits & Systems for Video Technology, 2014, 24(2):211–223.

[21] Gui J, Tao D, Sun Z, Luo Y, You X, Tang Y Y. Group sparse multiview patch alignment framework with view consistency for image classification. IEEE Transactions on Image Processing A Publication of the IEEE Signal Processing Society, 2014, 23(7):3126–37. 10.1109/TIP.2014.2326001 24860033

[22] Wang D, Nie F, Huang H. Feature Selection via Global Redundancy Minimization. IEEE Transactions on Knowledge & Data Engineering, 2015, PP(99):2743–2755.

[23] Gui J, Sun Z, Jia W, Jia W, Hu R, Lei Y, Ji S. Discriminant sparse neighborhood preserving embedding for face recognition[J]. Pattern Recognition, 2012, 45(8):2884–2893.

[24] Cai X, Nie F, Huang H. Exact top-k feature selection via l 2,0 -norm constraint// International Joint Conference on Artificial Intelligence, Beijing, China, August. 2013.

[25] Gui J, Wang S L, Lei Y K. Multi-step dimensionality reduction and semi-supervised graph-based tumor classification using gene expression data. Artificial Intelligence in Medicine, 2010, 50(3):181–191. 10.1016/j.artmed.2010.05.004 20599367

[26] Nie F, Huang H, Cai X, Ding C. Efficient and Robust Feature Selection via Joint ℓ2, 1-Norms Minimization// Advances in Neural Information Processing Systems 23:, Conference on Neural Information Processing Systems 2010. Proceedings of A Meeting Held 6–9 December 2010, Vancouver, British Columbia, Canada. 2010:1813–1821.

[27] Wasikowski M, Chen XW. Combating the small sample class imbalance problem using feature selection. IEEE Transactions on Knowledge and Data Engineering. 2010;22(10):1388–13.

[28] Forman G. An Extensive Empirical Study of Feature Selection Metrics for Text Classification. Journal of Machine Learning Research. 2003;3(2):1289–17.

[29] Liu T, Liu S, Chen Z. An evaluation on feature selection metrics for text clustering. Proc. International Conference on Machine Learning; 2003 Aug; Washington. DC, United States.

[30] Trang DD. New result in multiracial traffic analysis and modeling, Hungary: Department of Telecommunication and Media Informatics. PhD thesis, Budapest University of Technology and Economics; 2000

[31] ‘A. W. Moore, Dataset’, http://www.cl.cam.ac.uk/research/srg/netos/nprobe/data/papers/sigmet-rics/index.html, accessed Aug 2013

[32] Zhong W, Raahemi B, Liu J. Learning on Class Imbalanced Data to Classify Peer-to-Peer Applications in IP Traffic using Resampling Techniques// Neural Networks, IEEE—INNS—ENNS International Joint Conference on. IEEE, 2009:3548–3554.

[33] He H, Che C, Ma F, Luo X, Wang J. Improve Flow Accuracy and Byte Accuracy in Network Traffic Classification// Advanced Intelligent Computing Theories and Applications. With Aspects of Artificial Intelligence Springer Berlin Heidelberg, 2008:449–458.

[34] Jin Y, Duffield N, Erman J, Haffner P, Sen S, Zhang Z L. A Modular Machine Learning System for Flow-Level Traffic Classification in Large Networks. Acm Transactions on Knowledge Discovery from Data, 2012, 6(1):1–34.

[35] Williamson C. Internet traffic measurement. IEEE Internet Computing. 2001;5(6):70–5.

[36] Jeffrey CM. Network locality at the scale of processes. ACM Transactions on Computer Systems. 1992;10(2):81–29.

[37] Street W N. A streaming ensemble algorithm (SEA) for large-scale classification// ACM SIGKDD International Conference on Knowledge Discovery & Data Mining. 2001:1152–1165.

[38] Wang H, Fan W, Yu P S, Han J. Mining Concept-Drifting Data Streams Using Ensemble Classifiers. Kdd, 2003:226–235.

[39] Chen H X, Zhao Y Y, Dong J Z. Estimation of Mobile Target Track Based on Angular Information. Electro-Optic Technology Application, 2009.

[40] Hall MA. Correlation-based feature selection for machine learning. PhD thesis, The University of Waikato, 1999.

[41] Witten I H, Frank E, Hall M A. Data Mining: Practical Machine Learning Tools and Techniques (Third Edition)// Data Mining: Practical Machine Learning Tools and Techniques. Morgan Kaufmann Publishers Inc. 2011:206–207.

[42] Wald R, Khoshgoftaar T M, Napolitano A. Stability of Filter- and Wrapper-Based Feature Subset Selection. 2013:374–380.

